# Evaluating Cell Processes, Quality, and Biomarkers in Pluripotent Stem Cells Using Video Bioinformatics

**DOI:** 10.1371/journal.pone.0148642

**Published:** 2016-02-05

**Authors:** Atena Zahedi, Vincent On, Sabrina C. Lin, Brett C. Bays, Esther Omaiye, Bir Bhanu, Prue Talbot

**Affiliations:** 1 Bioengineering Department, University of California, Riverside, California, United States of America; 2 Electrical and Computer Engineering Department, University of California, Riverside, California, United States of America; 3 Center for Research in Intelligent Systems, University of California, Riverside, California, United States of America; 4 Department of Cell Biology and Neuroscience, University of California, Riverside, California, United States of America; 5 UCR Stem Cell Center and Core, University of California, Riverside, California, United States of America; 6 Psychology Department, University of California, Riverside, California, United States of America; University of Kansas Medical Center, UNITED STATES

## Abstract

There is a foundational need for quality control tools in stem cell laboratories engaged in basic research, regenerative therapies, and toxicological studies. These tools require automated methods for evaluating cell processes and quality during *in vitro* passaging, expansion, maintenance, and differentiation. In this paper, an unbiased, automated high-content profiling toolkit, StemCellQC, is presented that non-invasively extracts information on cell quality and cellular processes from time-lapse phase-contrast videos. Twenty four (24) morphological and dynamic features were analyzed in healthy, unhealthy, and dying human embryonic stem cell (hESC) colonies to identify those features that were affected in each group. Multiple features differed in the healthy versus unhealthy/dying groups, and these features were linked to growth, motility, and death. Biomarkers were discovered that predicted cell processes before they were detectable by manual observation. StemCellQC distinguished healthy and unhealthy/dying hESC colonies with 96% accuracy by non-invasively measuring and tracking dynamic and morphological features over 48 hours. Changes in cellular processes can be monitored by StemCellQC and predictions can be made about the quality of pluripotent stem cell colonies. This toolkit reduced the time and resources required to track multiple pluripotent stem cell colonies and eliminated handling errors and false classifications due to human bias. StemCellQC provided both user-specified and classifier-determined analysis in cases where the affected features are not intuitive or anticipated. Video analysis algorithms allowed assessment of biological phenomena using automatic detection analysis, which can aid facilities where maintaining stem cell quality and/or monitoring changes in cellular processes are essential. In the future StemCellQC can be expanded to include other features, cell types, treatments, and differentiating cells.

## Introduction

Human pluripotent stem cells (hPSC) have enormous potential for enhancing our understanding of human prenatal development, modeling diseases-in-a-dish, treating patients with degenerative diseases, and evaluating the effects of drugs and environmental chemicals on cells that model human embryos and fetuses [1–3]. In each of these applications, there is a foundational unmet need for technology to non-invasively monitor the quality of hPSC during passaging, expansion, growth, experimentation, and differentiation [4, 5]. Ideally such tools should be rapid, non-invasive, resource saving, and non-biased. Video bioinformatics, which involves mining data from video images using algorithms that speed analysis and eliminate human bias, offers a solution to this problem and can be used to produce high quality software for stem cell applications [6–13].

Prior applications of video bioinformatics tools have successfully identified pluripotent stem cell colonies based on colony morphology [14], thereby speeding induced pluripotent stem cell (iPSC) derivation and reducing cost. Another study applied image processing software to fluorescent videos to identify iPSC after reprogramming [15], and a video bioinformatics method was developed to identify in vitro fertilized human embryos that will progress to blastocysts by 2 days after fertilization [16]. A recent report used phase-contrast video segmentation to generate lineage trees of neural stem cells using cell location, shape, movement, and size [17]. We previously developed in-house video segmentation tools to analyze single hESC and small colonies [8–11]. In a toxicological application of video bioinformatics using CL-Quant software [18], cigarette smoke treatment altered hESC colony growth (area) and health [7]. While the above studies looked at single endpoints, multiple features related to cellular processes and health can be extracted from video data thereby enhancing the depth of analysis and providing data on the kinetics of each endpoint. However, no software currently exists for automatic detection of pluripotent stem cell processes and quality in culture.

It is highly desirable to be able to multiplex multiple endpoints from a single experiment. The purpose of this study was to develop a high-content profiling software platform, StemCellQC, to automatically identify cell processes affected by culture/treatment and to classify the health of individual hESC colonies based on features extracted from phase contrast microscope video data. The method automatically segments the input colonies (non-labeled phase contrast images), extracts relevant novel features for each colony, utilizes the changes in features over time to identify cell processes that are affected by treatment, and statistically classifies healthy and unhealthy/dying colonies. StemCellQC’s feature analysis and classification system provide an effective method to evaluate pluripotent stem cell colony processes and quality before use in experiments or clinical applications. Because hESC model the epiblast cells of embryos [19], which if harmed can lead to embryonic death or development of congenital defect(s), StemCellQC has the potential to be used as a novel technology to identify toxicants or drugs that could affect cellular processes in young embryos.

## Materials and Methods

### Overall Design of the System

The overall design of the system will be discussed in Results (S1 Fig). Each component is presented in the following Methods section.

### Culture and Collection of hESC Videos

H9 hESC, purchased from WiCell Stem Cell Institute (Madison, Wisconsin), were maintained on Matrigel coated 6-well plates in mTeSR complete medium (Stem Cell Technologies, Vancouver, Canada) in a 37°C incubator with 90% humidity and 5% CO_2_ [7,20]. When cultures reached 80–85% confluency, they were detached using Accutase (eBiosciences, San Diego, CA) for 1 minute and used in experiments.

For live cell imaging in the BioStation CT (Nikon Instruments, Melville NY), hESC colonies were plated at 25–30% confluency and allowed to attach for 24 hours. To create groups of colonies that were healthy, unhealthy and dying, hESC were treated with Marlboro Red cigarette smoke solutions as described previously [7,21]. Sidestream smoke (SS) was used at a dose of 0.1 puff equivalent (PE), where 1 PE is the amount of smoke that dissolves in 1 ml of medium in 60 sec. The 0.1PE concentration of SS smoke has an estimated concentration of nicotine of 0.2 μg/ml [22,23], which is within the estimated tissue range of passive smokers [21].

All imaging was done using a 10x phase contrast objective in the BioStation CT using automatic Z-focus; cells were not stained, labeled, or genetically modified thereby permitting non-invasive analysis of cells. StemCellQC was tested on different magnifications (4x and 20x), and performs well. The dataset for feature analysis was made up of 34 videos of individual hESC colonies. 23 colonies were treated for 48 hours with sidestream cigarette smoke, while 11 control colonies were incubated in culture medium only.

### Development and Use of StemCellQC Software

StemCellQC was written and developed with MATLAB 2015a programming environment. The MATLAB source code, a stand-alone executable version of this algorithm, and supplied test data are available online at http://vislab.ucr.edu/SOFTWARE/software.php. Scqc_multi.m is the main program of the code and requires the following MATLAB toolboxes: Statistics and Machine Learning, Bioinformatics, System Identification, Image Processing, and Model-Based Calibration. The standalone alone executable requires the installation of the 64-bit version of MATLAB Runtime R2015a (8.5) available at http://www.mathworks.com/products/compiler/mcr/.

### Categorization of hESC as Healthy, Unhealthy or Dying

Before the StemCellQC software is run, a training dataset was collected. Categorization of colonies as healthy, unhealthy, or dying by the end of 48 hours was provided by experts in culturing hESC and was based on biological observations listed in a decision tree (S2 Fig). Categorization was used to validate the program’s predictions of colony health.

### Video Segmentation

An edge-based method and a region-based method were used to segment colonies in video images (frames). The edge-based method convolves the Sobel edge operator with the image to produce a gradient magnitude image and a gradient direction image. All gradient magnitudes below a calculated threshold were ignored and the rest were used as edges. This calculated threshold was automatically computed by the “edge” function in the Image Processing Toolbox from MATLAB. The edges in the image were dilated using two line structuring elements (vertical and horizontal) of three pixel lengths to merge connected regions of the colony. Connected components were then filled and smoothed with image erosion using a diamond structuring element of one pixel radius to produce the segmentations. Segmented objects that are smaller than a user specified threshold, 3000 pixels in our case, were removed [24].

The Otsu’s region-based method for segmentation [25,26], which was used to compute the solidity feature, is the ratio of the colony area divided by the area of the convex hull. The convex hull can be visualized as the shape enclosed by a rubber band stretched around a region-of-interest (ROI) [27]. Otsu’s method produces a slightly larger segmented boundary which is smoother than the edge-based method. The main purpose of using solidity was to detect dead cells that were extruded from the stem cell colonies. Edge-based segmentation provides tight edge boundaries, which does not include the dead cells that are in the process of being expelled from the colony; whereas, Otsu’s “larger” segmentation includes the dead cells. The concave regions of the segmentation that are produced by the dead cells affect the sensitivity of solidity. Therefore, solidity changes (primarily due to dead cells) are more pronounced when using Otsu’s method. This larger boundary results in a larger convex hull which increases the sensitivity of the solidity feature. This allowed for better distinction between the peaks and valleys in the solidity plot.

For the region-based method, initially frames were smoothed to remove a small amount of noise using a 3x3 Gaussian filter. Next, Ostu’s thresholding-based method was used to separate the pixels into the background and foreground by finding the optimal threshold for segmenting an image [25,26]. Connected components were then found in the binary image. A morphological open operation (used to open gaps between loosely connected objects) was performed on the binary image to disconnect loosely connected pixels in the foreground. This is carried out by first eroding an object of interest (a connected component) and then dilating the output with a structuring element. To disconnect the objects, a circle with a radius of 12 pixels worked the best, and it was kept fixed for all the experiments. For both erosion and dilation, every pixel in the object was individually probed by the structuring element. The end result was a set of filtered connected components. Any holes in this region were filled, and the final ROIs were used to extract features.

### Validation of Segmentation

The accuracy of segmentation was determined by manually segmenting hESC colonies using ImageJ and comparing the area and perimeter values to those obtained from the automatic segmentation (S3 Fig).

### Feature Extraction

Features based on appearance, morphology, and dynamics were extracted from segmented colonies. Dynamic features were obtained from morphological features by computing the rate of change of a feature over time. For example, to segment the protrusions extending from hESC, the main body of the colony (obtained using a morphological open algorithmic operation) was subtracted from the total colony segmentation. Also, the bright-to-total area ratio is the number of bright pixels divided by the area. Bright pixels were found by first computing a histogram of the intensities in the segmented colony to acquire the mean (μ) and standard deviation (σ). Then, a range of intensities (lower bound threshold = μ + 3 σ, upper bound threshold = μ + 6 σ) that best described dead cells in colonies were taken as the bright pixels. A complete list of features and their definitions is provided in S9 Fig.

### Identification of Key Features

Both biologically-based feature selection and statistical-based feature selection were used to identify those features that provided information on affected cellular processes and to distinguish healthy, unhealthy, and dying colonies. In addition, all the features were exhaustively applied to the classifier.

**Fig 1:**
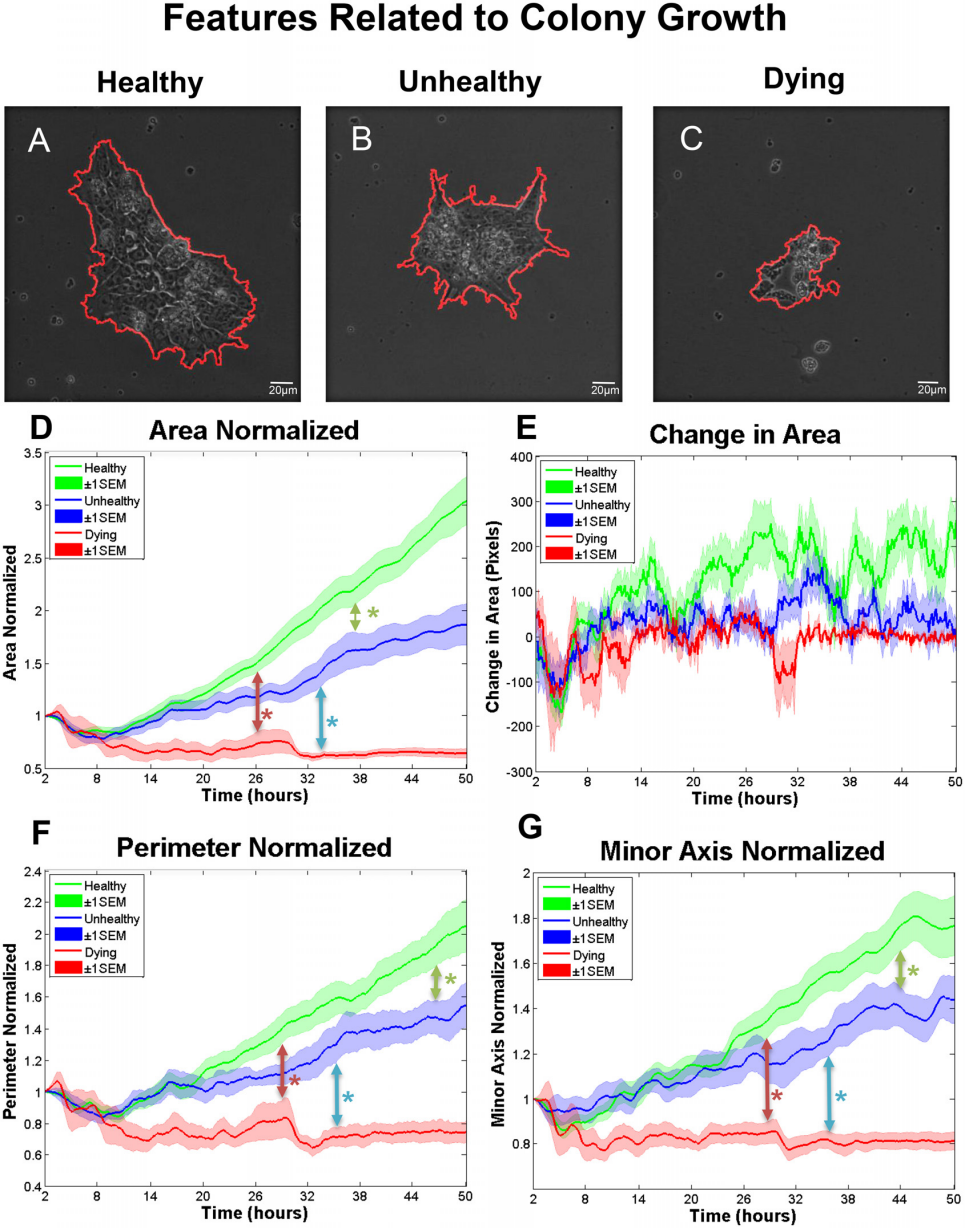
Features related to hESC colony growth. The outline of segmentation for a healthy (A), unhealthy (B), and a dying colony (C) at the last recorded frame. (D) Area normalized to the first time point for colonies that were healthy, unhealthy, and dying. Colonies first became significantly different by 2-way ANOVA at 37.6 hours for healthy vs unhealthy (green arrow), at 33.5 hours for unhealthy versus dying (blue arrow), and at 26.2 hours for healthy versus dying colonies (red arrow). (E) Change in area over time showing second contraction of dying colonies at 30–32 hours. (F) Perimeter over time normalized to the first time point for colonies that were healthy, unhealthy, and dying. Colonies first became significantly different by 2-way ANOVA at 46.6 hours for healthy versus unhealthy groups (green arrow), at 35.4 hours for unhealthy versus dying groups (blue arrow), and at 28.9 hours for healthy versus dying groups (red arrow). (G) Minor axis normalized to the first time point for colonies that were healthy, unhealthy, and dying. Colonies first became significantly different by 2-way ANOVA at 44.3 hour for healthy versus unhealthy groups (green arrow), at 36.5 hour for unhealthy versus dying groups (blue arrow), and at 28.9 hour for healthy versus dying group (red arrow). Number of colonies per group = 16 healthy, 12 unhealthy, 6 dying. Data are plotted as means ± SEM for each group. Arrows indicate first values that differed significantly from the control by 2-way ANOVA (* = P < 0.05).

**Fig 2:**
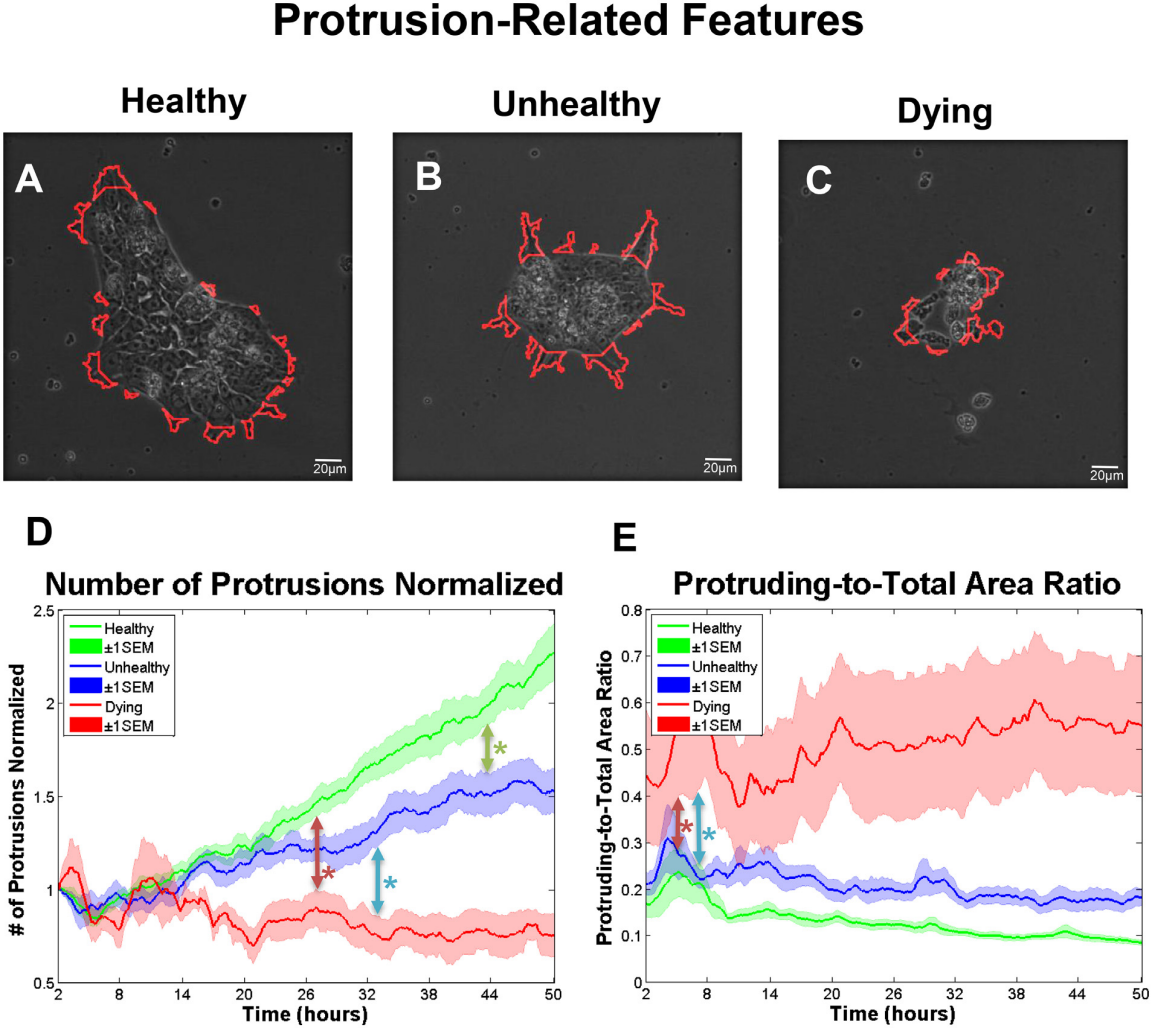
Surface protrusions on colonies can be used to study cell morphology and growth. (A) Segmentation of protrusions (red outline) for a healthy colony (A), unhealthy colony (B), and dying colony (C) at the last recorded time frame. (D) Number of protrusions over time normalized to the initial time point for healthy, unhealthy, and dying colonies. Colonies first became significantly different by 2-way ANOVA at 43 hours for healthy versus unhealthy groups (green arrow), at 33.3 hours for unhealthy versus dying groups (blue arrow), and at 27.1 hours for healthy versus dying group (red arrow). (E) Protruding-to-total area ratio for healthy, unhealthy, and dying colonies. Colonies first became significantly different by 2-way ANOVA at 6.8 hours for unhealthy versus dying groups (blue arrow), and at 4.8 hours for healthy versus dying groups (red arrow). Number of colonies per group = 16 healthy, 12 unhealthy, 6 dying. Data are plotted as means ± SEM for each group. Arrows indicate first values that differed significant from the control by 2-way ANOVA (* = P < 0.05).

**Fig 3:**
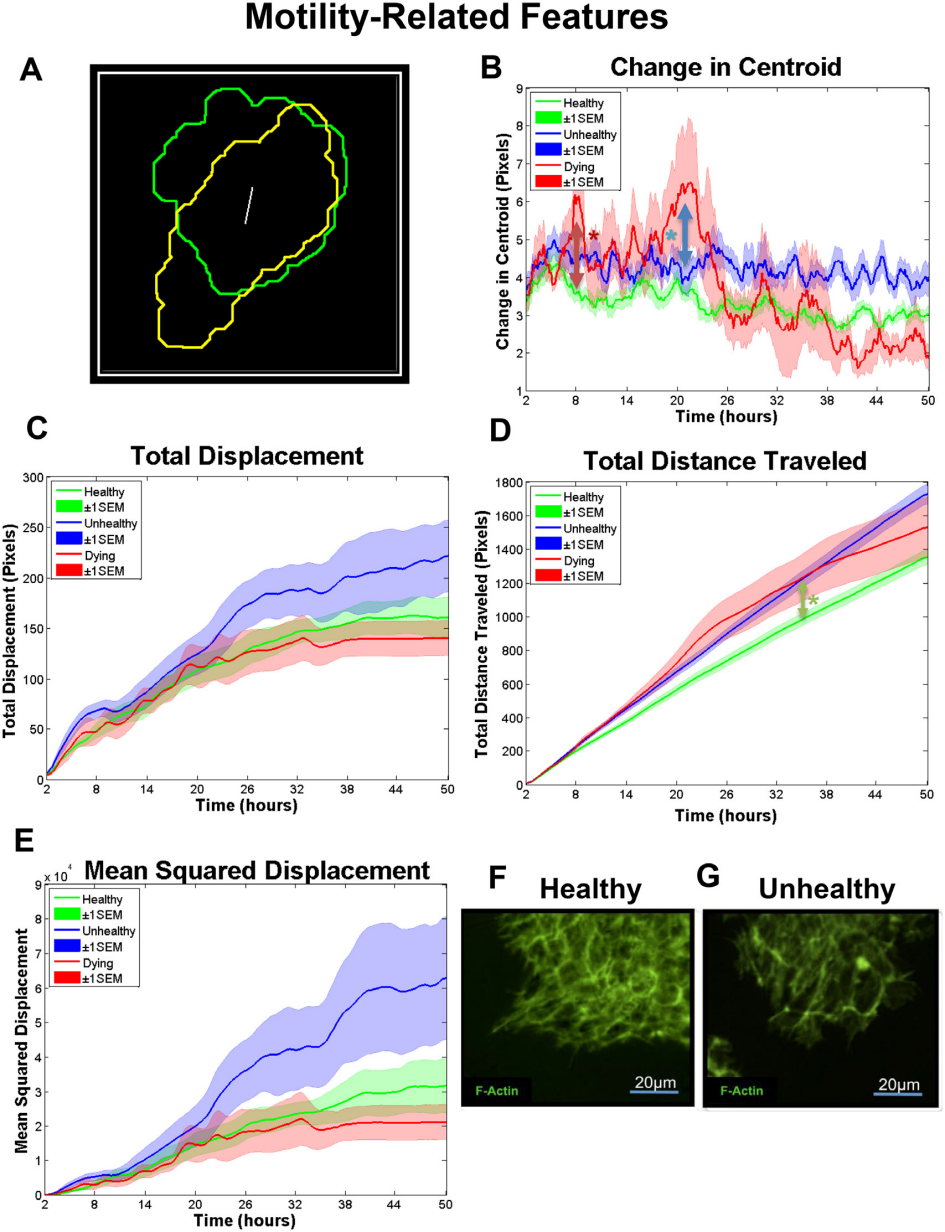
Features related to hESC colony motility. Extracted contour of a healthy colony at 16 hours (yellow line) and 24 (green line) hrs. The distance between the centroids is indicated by the white line. (B) Change in centroid over time for healthy, unhealthy, and dying colonies. Colonies first became significantly different by 2-way ANOVA at 7.7 hours for healthy versus dying groups (red arrow), and at 20.6 hours for unhealthy versus dying groups (blue arrow). (C) The total displacement for healthy, unhealthy, and dying colonies. (D) The total distance traveled for healthy, unhealthy, and dying colonies. Colonies first became significantly different by 2-way ANOVA at 34.7 hours for healthy versus unhealthy groups (green arrow). (E) The mean squared displacement (MSD) for healthy, unhealthy, and dying colonies. (F-G) Localization of actin microfilaments in a healthy (F) and an unhealthy (G) colony which had fewer microfilaments than the untreated control. Number of colonies per group = 16 healthy, 12 unhealthy, 6 dying. Data are plotted as means ± SEM for each group. Arrows indicate first values that differed significant from the control by 2-way ANOVA (* = P < 0.05).

**Fig 4:**
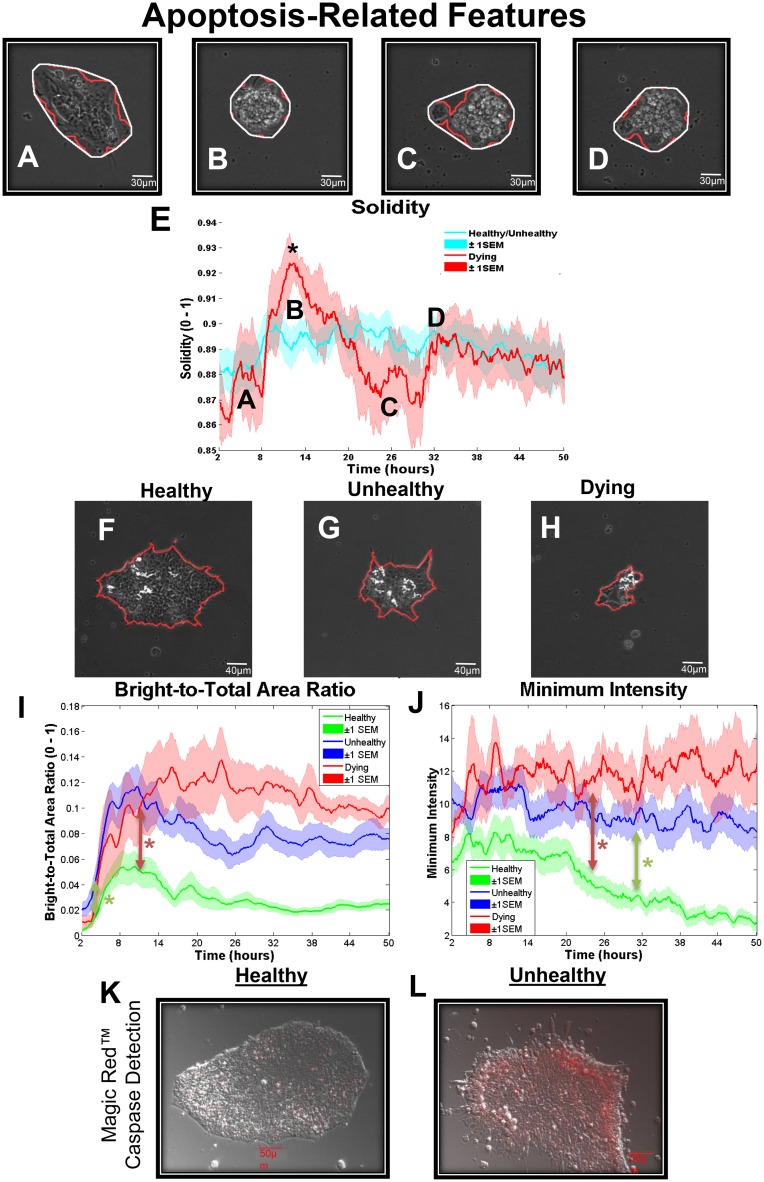
Features related to cell death. (A-D) Frames representing the beginning of the video (A), the highest and lowest solidity values respectively (B and C), and the time of death of dying colonies (D). E) Solidity values over time for healthy/unhealthy (blue) versus dying colonies (red). Colonies that eventually died are distinguished by a large peak in solidity between 8–24 hours. A one-tailed independent sample t-test at 12 hours revealed that the two groups were significantly different (P = 0.0285). (F-H) White regions on top of hESC colonies (outlined in red) represent dead cells, shown at the end of recording for a healthy colony (F), unhealthy colony (G), and dying colony (H). (I) Bright-to-total area ratio over time for healthy, unhealthy, and dying colonies. Colonies first became significantly different by 2-way ANOVA at 4 hours for healthy versus unhealthy groups (green arrow), and at 11.5 hour for the healthy versus dying groups (red arrow). (J) Minimum intensity values for healthy, unhealthy, and dying colonies. Colonies first became significantly different by 2-way ANOVA at 31.5 hour healthy versus unhealthy groups (green arrow), and at 24 hours for healthy versus dying groups (red arrow). (K-L) A healthy (K) and an unhealthy (L) colony incubated with Magic Red to identify activated caspases 3&7. Number of colonies per group = 16 healthy, 12 unhealthy, 6 dying. Data are plotted as means ± SEM for each group. Arrows indicate first values that differed significant from the control by 2-way ANOVA (* = P < 0.05).

For biologically-based feature selection, plot observations over time can be interpreted by the user. A subset of features are shown as graphical plots in Figs 1–4. From the feature plots, the user can observe the non-overlapping standard error of the means (SEMs) to get a good indication of groups that are significantly different from each other. For a more rigorous statistical analysis, two-way ANOVAs with the Bonferroni post-test were performed to identify those features that were significantly different in the plotted data. For the solidity feature, a one-tailed independent samples t-test was used to determine if significant differences existed between means of healthy/unhealthy versus /dying colonies at 12 hours.

Statistical-based methods are useful in cases where the graphs for features may not reveal obvious effects, and they are good starting points to identify combinations or subsets of useful features. Filter methods which select variables regardless of the classification model are preferable for StemCellQC because of the use of multiple classifiers. 11 feature selection algorithms (10 methods from the Feature Selection @ Arizona State University toolbox [28]) and quadratic programming feature selection [29] were run on our dataset. These methods include Correlation-based Feature Selection (CFS) [30], Chi Square (Chi2) [31], Fast Correlation-based Filter (FCBF) [32], Fisher 9 [33], Gini Index 16 [34], InfoGain 6 [35], Sparse Multinomial Logistic Regression (SBMLR 3) [36], t-test [37], Kruskal Wallis [38], and Minimal-Redundancy-Maximal-Relevance [39]. The inputs for the feature selection algorithms are the average slope of each feature. The slope (incremental difference) is computed for each pair of adjacent frames for individual features. Next, the mean of these slopes is calculated for all 24 features. These features individually or in groups can be used to train the classifier.

### Correlation of Key Features to Cellular Processes

**Fig 5:**
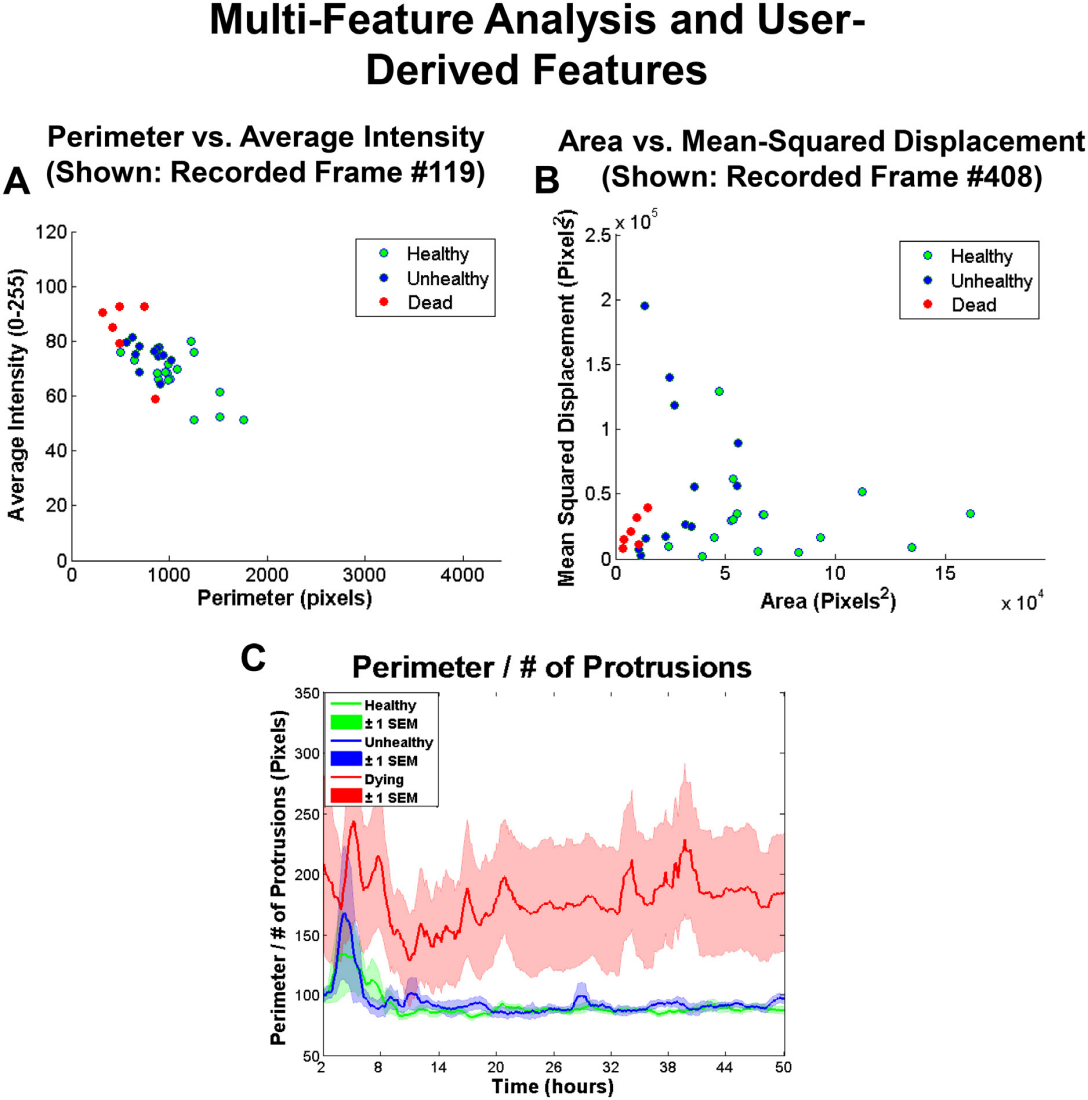
Two-Feature Plot Analysis. (A) Average intensity compared to perimeter running plot shown at approximately at 16 hours of incubation for all individual healthy (green), unhealthy (blue), and dying (red) hESC colonies. (B) Mean-squared displacement compared to area running plot shown at approximately 16 hours for all individual healthy (green), unhealthy (blue), and dying (red) hESC colonies. (C) User derived equation (perimeter divided by number of protrusions) plotted for healthy, unhealthy, and dying colonies.

Key features were interpreted to identify cellular processes such as growth, motility, and apoptosis, which differed in the healthy, unhealthy and dying groups. Also, two-feature plots were constructed to detect correlations between features and identify temporal patterns over time (Fig 5, S1 and S2 Videos).

### Classification as Healthy or Unhealthy/Dying

To automatically classify the dataset, all features measurements were first normalized with maximum-minimum normalization. 410 frames (collected over a 48 hour period) multiplied by 24 features results in a 9840 feature space. Therefore, in order to reduce the number of dimensions, the input value to the classifier was the mean slope of the data. Classes used by the classifier were healthy and unhealthy (the unhealthy group included dying colonies which were not classified separately since this group contained only 6 colonies).

To test the ability of the features to distinguish healthy versus unhealthy/dying colonies, several biologically selected features and additional features selected by 11 statistical methods were used to train three classifiers: (1) support vector machines (SVM), (2) K-nearest neighbor (KNN), and (3) naïve Bayes [33]. SVM uses the training data to create a boundary in multi-dimensional space, which can be used to classify future data samples. KNN takes a test sample and compares it to the K-nearest training samples in a multi-dimensional space. The KNN algorithm was used with k = 3 (the 3 closest neighbors to the sample). A majority vote is taken by these neighbors and used as a label for the test sample. Naïve Bayes is a probabilistic classifier based on Bayes’ theorem that uses strong assumptions that features are independent from one another. An exhaustive test using all possible combinations of features was performed to determine the best classification results using combination of features.

A summary of the classification results using single features, combinations of features, and statistically determined features are shown in Tables 1–4 in the Results section.

**Table 1:** Classification Results Using 48 Hours of Video.

48 Hours	*Classification Techniques		
Single Features	SVM	K-NN, k = 3	Naïve Bayes
1) Area	94.12 ± 0.00	94.12 ± 0.00	94.00 ± 0.91
2) Number of Protrusions	90.71 ± 1.35	96.06 ± 1.32	91.24 ± 0.65
3) Total Distance Travelled	84.24 ± 1.20	74.06 ± 1.48	84.88 ± 1.01
**Combination of Features**			
1) Area, Orientation, Num. of Protrusions	94.12 ± 0.00	94.71 ± 1.15	94.12 ± 0.00
2) Num. of Protrusions, Min. Intensity	**97.06 ± 0.00**	**97.06 ± 0.00**	**96.47 ± 1.15**
3) Major Axis, Minor Axis, Change in Centroid	93.53 ± 1.57	92.94 ± 1.62	90.00 ± 1.27
**Feature Selection Methods**			
**CFS	91.76 ± 1.32	96.47 ± 1.32	91.76 ± 1.32
***Chi Square	91.76 ± 0.00	91.76 ± 0.00	95.29 ± 1.61
****QPFS	91.76 ± 1.32	94.12 ± 3.60	91.76 ± 2.46

*Classification of colonies as healthy or unhealthy using three different classification techniques: SVM, KNN, and Naive Bayes.

**CFS selected the following features: Area, Number of Protrusions, and Change in Area.

***ChiSquare selected the following features: Area, Number of Protrusions, and Major Axis Length

****Quadratic Programming Feature Selection selected the following features: Total Distance Travelled, Major Axis Length, Minimum Radius.

**Table 2:** Classification Results Using 36 Hours of Video.

36 Hours	*Classification Techniques		
Single Features	SVM	K-NN, k = 3	Naïve Bayes
1) Area	85.18 ± 1.25	88.00 ± 1.49	85.18 ± 1.49
2) Number of Protrusions	81.94 ± 1.10	73.65 ± 1.72	81.00 ± 1.34
3) Bright Area Ratio	81.76 ± 3.22	85.29 ± 0.00	76.47 ± 2.08
**Combination of Features**			
1) Area, Min. Radius, Num. of Protrusions, Change in Area, Change in Perimeter	96.47 ± 1.32	94.71 ± 1.32	94.71 ± 1.32
2) Area, Min. Radius, Change in Area, Change in Perimeter	95.88 ± 1.61	92.94 ± 1.61	95.88 ± 1.61
3) Area, Avg. Radius, Change in Area, Change in Perimeter	95.29 ± 1.61	92.94 ± 1.61	95.88 ± 2.63
**Feature Selection Methods**			
**CFS	91.18 ± 2.08	88.24 ± 2.08	90.00 ± 1.61
***SMBLR	85.88 ± 1.32	91.76 ± 1.32	90.59 ± 1.32
****FCBF	86.47 ± 1.61	87.65 ± 1.32	92.94 ± 1.61

*Classification of colonies as healthy or unhealthy using three different classification techniques: SVM, KNN, and Naive Bayes.

**CFS selected the following features: Area, Perimeter, Minor Axis Length, Minimum Intensity, Bright Area Ratio, Number of Protrusions, Change in Perimeter, Total Distance Travelled.

***SMBLR selected the following features: Number of Protrusions, Change in Perimeter, Minor Axis Length, Bright Area Ratio, Total Distance Travelled.

****FCBF selected the following features: Area, Perimeter, Minimum Intensity, Bright Area Ratio, Change in Perimeter, total Distance Travelled.

**Table 3:** Classification Results Using 24 Hours of Video.

24 Hours	*Classification Techniques		
Single Features	SVM	K-NN, k = 3	Naïve Bayes
1) Area	83.35 ± 1.49	74.94 ± 2.00	82.47 ± 1.37
2) Number of Protrusions	75.29 ± 1.85	67.41 ± 1.95	71.35 ± 1.51
3) Perimeter	75.29 ± 4.36	73.53 ± 2.08	75.29 ± 1.61
**Combination of Features**			
1) Area, Orientation, Num. of Protrusions, Change in Perimeter, Change in Centroid	83.53 ± 1.61	91.17 ± 0.00	88.82 ± 1.31
2) Area, Avg. Radius, Change in Perimeter, Change in Centroid	88.24 ± 2.08	83.53 ± 1.61	90.59 ± 1.32
3) Orientation, Bright Area Ratio, Change in Perimeter, Change in Centroid	87.65 ± 1.32	88.24 ± 2.94	84.12 ± 1.61
**Feature Selection Methods**			
*CFS	84.71 ± 1.32	72.35 ± 4.46	78.24 ± 1.61
**SBMLR	78.24 ± 5.73	69.42 ± 3.35	83.53 ± 3.35
***FCBF	84.71 ± 1.32	72.35 ± 4.46	78.24 ± 1.61

*Classification of colonies as healthy or unhealthy using three different classification techniques: SVM, KNN, and Naive Bayes.

**CFS selected the following features: Area, Minor Axis Length, Bright Area Ratio, Change in Perimeter, Total Distance Travelled.

***SMBLR selected the following features: Area, Orientation, Number of Protrusions, Total Distance Travelled.

****FCBF selected the following features: Area, Minor Axis Length, Bright Area Ratio, Change in Perimeter, Total Distance Travelled.

**Table 4:** Classification Results Using 12 Hours of Video.

12 Hours	*Classification Techniques		
Single Features	SVM	K-NN, k = 3	Naïve Bayes
1) Bright Area Ratio	67.06 ± 1.32	54.12 ± 1.61	64.70 ± 2.94
2) Total Distance Travelled	71.76 ± 2.63	65.29 ± 2.46	61.18 ± 3.83
3) Change in Centroid	64.71 ± 0.00	57.06 ± 3.35	67.06 ± 2.46
**Combination of Features**			
1) Max. Radius, Ratio of Protrusion Area, Change in Area, Change in Centroid	74.12 ± 2.46	75.88 ± 1.31	79.41 ± 3.60
2) Eccentricity, Min. Radius, Ratio of Protrusion Area, Change in Perimeter, Change in Centroid	71.18 ± 3.83	78.24 ± 3.35	78.24 ± 1.61
3) Min. Radius, Max Intensity, Change in Centroid	71.18 ± 2.46	80.59 ± 2.63	73.53 ± 0.00
**Feature Selection Methods**			
*CFS	71.76 ± 2.63	65.29 ± 2.46	61.18 ± 3.83
**SBMLR	67.65 ± 2.94	77.06 ± 2.46	64.12 ± 3.22
***FCBF	71.76 ± 2.63	65.29 ± 2.46	61.18 ± 3.83

*Classification of colonies as healthy or unhealthy using three different classification techniques: SVM, KNN, and Naive Bayes.

**CFS selected the following features: Total Distance Travelled.

***SMBLR selected the following features: Change in Area, Total Distance Travelled.

****FCBF selected the following features: Total Distance Travelled.

### Classification Validation

The classification experiments were run with 10-fold cross validation where the dataset was partitioned into 10 parts. The 34 videos gave six partitions containing three videos each and four partitions containing four videos each. A 10-fold partition of the training data was used, which allowed for computation of a standard deviation of the results. One part was used as the test data once, while the other nine parts were used as training data. The partitions were randomized, and this process was repeated with 5 random permutations of the data. A percentage of correctly classified samples was calculated for each permutation by comparison to the manual labels. The classification results were then used to find the mean and standard deviation.

### Molecular Validation

Apoptotic activity was detected using the Magic Red Caspases 3&7 Detection FLICA Kit (Immunochemistry Technologies, LLC, Bloomington, MN) as described previously [7]. Fluorescent staining of F-actin was performed using a phalloidin-Alexa Fluor 488 conjugate (Invitrogen, Carlsbad, CA) diluted 1:200 in 1% goat serum in phosphate buffered saline. hESC colonies in chamber slides were fixed using 4% paraformaldehyde for 10 minutes, incubated in blocking solution (3% goat serum in PBS) at room temperature for 1 hour, washed 5 times, and incubated in phalloidin-Alexa Fluor 488 for 1 hour at room temperature. Samples were mounted using Vectashield with DAPI (Vector Laboratories, Burlingame, CA) and imaged with a Nikon Eclipse Ti fluorescent microscope (Nikon, Melville, NY).

## Results

### Feature Analysis

Features were analyzed graphically to identify those that differed in the healthy, unhealthy, and dying groups. Sets of affected features were then grouped according to the biological processes they represented (morphology, growth, motility, death) (Figs 1–4, S4 Fig). The classifiers were run with 48, 36, 24, and 12 hours of video to show their effectiveness at different time points. For all durations, all 24 features were run singularly through the classifiers and the ones with the highest accuracy are shown in Tables 1–4 (Single Features). Additionally, exhaustive searches for combinations of up to 5 features were run to identify the most accurate results (Table 1 Combination of Features). Lastly, the best results from the 11 existing feature selection algorithms are also shown in Tables 1–4 (Feature Selection Methods).

### Features Related to Colony Growth as Biomarkers of hESC Health

Extracted features related to colony growth (area, perimeter, minor axis, protrusions) were evaluated in healthy, unhealthy, and dying colonies. Area (the total number of pixels inside a segmented colony; S5 Fig) differed in healthy, unhealthy and dying colonies (Fig 1A–1E). When area was normalized to the initial time point to account for variability in the starting size, all groups displayed an initial contraction which decreased area and lasted about 8 hours (Fig 1D). Contraction was likely caused by changes in temperature/CO_2_ during transfer to the BioStation. After contraction, healthy colonies displayed a steady increase in area until the end of recording, while unhealthy colonies grew at a slower and variable rate. Both groups followed a similar trend up to 16 hours, after which healthy and unhealthy growth rates deviated and become distinguishable by about 24 hours. Dying colonies could be distinguished from the healthy and unhealthy groups by about 10 hours when growth rates for the dying group clearly diverged. At 30 hours, dying colonies underwent a second contraction leading to a sharp decrease in area (Fig 1D and 1E). These colonies were interpreted to be dead based on this pronounced decrease in size and shedding of dead cells.

Perimeter, defined as the number of pixels constituting the colony periphery (red outline in Fig 1A–1C and 1F; S5 Fig), provided additional information about colony growth. There was divergence in the perimeters of healthy and unhealthy colonies at approximately 25 hours (Fig 1F), after which the rate of change in perimeter for unhealthy colonies slowed until about 33 hours when it underwent a growth spurt that lasted 3 hours. The dying colonies diverged from the other two groups at 8 hours and had an abrupt decrease in size at 30 hours, as was seen with area.

Minor axis (smaller axis of an ellipse fitted to a colony) (S6 Fig) was affected in unhealthy/dying colonies (Fig 1G). After 23 hours, healthy colonies showed a steep increase in minor axis, suggesting that once healthy colonies reach a critical size, they have a less-elongated morphology.

Protrusions are dynamic cell processes that extend off colonies and take a variety of shapes (Fig 2A–2C; S6 Fig). They allow colonies to attach, spread, and migrate, [40,41]. The number of protrusions increased on healthy and unhealthy colonies and decreased on dying colonies during incubation (Fig 2D). The protruding-to-total area ratio, which is defined as the ratio of protrusion area divided by total colony area, had an inverse relationship with colony growth. Protrusion area decreased gradually in healthy and unhealthy groups, but increased slightly in the dying group (Fig 2E).

### Colony Motility

The change in centroid feature allowed tracking of stem cell colony movement. This feature is determined by finding the centroid of each colony and calculating the distance between two successive frames (S7 Fig). Outlines of a hESC colony at two times and the change in centroids are shown in Fig 3A. Change in centroid oscillations were smaller in the healthy and unhealthy groups than in the dying group (Fig 3B). The unhealthy and healthy groups were similar in the magnitude of their oscillations, but overall motility was less in the healthy colonies, probably because the larger sized healthy colonies required more energy and coordination for directed movement. After a certain area was reached, the center of the healthy colonies moved very little as the colony continued to expand. It is also possible that smoke stimulated motility in unhealthy hESC to facilitate escape from exposure. The dying colonies displayed erratic motility and showed a significant decrease in movement after 20 hours as they were approaching death. Movements detected after death (30 hours) are due to slight segmentation differences between frames.

Total displacement detected how far a colony moved from its original starting point (Fig 3C; S7 Fig), while total distance traveled is the sum of the entire trajectory of movement (Fig 3D; S7 Fig). These features revealed information on the pattern of travel. Dying colonies traveled more up to 30 hours (when they died) than the other two groups, but their displacement was low indicating that they moved erratically near their original starting point. Unhealthy colonies moved further from their point of origin and travelled a longer total distance than healthy colonies. Both the healthy and unhealthy colonies displayed remarkably little variance in total distance travelled (Fig 3D).

The mean squared displacement (MSD) feature measures Brownian motion [42] and can be used to study cellular migration [43]. MSD is defined by the equatio: *MSD*(*t*) = ([*x*(*t*+*t0*) − *x*(*t0*)]^2^+[*y*(*t*+*t0*) − *y*(*t0*)]^2^), where MSD (t) can be approximated as ~ t^β(t)^. The logarithmic derivative exponent β can be used to determine the particular mode of motility, with β > 1 indicating super-diffusive movement, a form of diffusion where the colonies occasionally undergo very long steps. β < 1 indicates sub-diffusive movement, defined as a tendency for the colonies not to diffuse due to trapping (inability to move). For Brownian motion, or a random walk, β is approximately 1. The MSD feature is robust because it uses the squared value of displacement, making it less sensitive to small fluctuations. The MSD plot shows a similar trajectory for all three groups up until about 11 hours (Fig 3E), after which the healthy colonies display Brownian motion (β = 1.04). For dying colonies, sub-diffusive motility (β = 0.21) was observed from 23–50 hours, consist with their death after 30 hours. The unhealthy group demonstrated sub-diffusive motility from 22–35 hours and 35–50 hours (β values = 0.76 and 0.52, respectively).

To investigate the molecular basis of the aforementioned effects on motility, F-actin was labeled with phalloidin-Alexa 488. Healthy colonies (Fig 3F) had a more robust actin cytoskeleton than unhealthy colonies (Fig 3G). Although F-actin was partially depolymerized by smoke treatment, there was sufficient functional F-actin in the treated colonies to allow colony movement. A decrease in F-actin may be linked to a decrease in the number of focal adhesions, which may facilitate motility in the unhealthy group [44,45]. Other studies have reported the inverse correlation between cell motility and polymerization state of the actin cytoskeleton [46].

### Solidity as Predictor of Apoptosis

As colonies became rounder or more convex, their solidity increased and approached 1. Fig 4A–4D show hESC colonies at different times with outlines of their segmentations (red lines) and convex hulls (white lines). Solidity, which measured convexity (Fig 4E; S8 Fig), identified colonies that were destined to die by 48 hours. Solidity for the healthy/unhealthy groups combined changed little during 48 hours (Fig 4E). These two groups were combined since solidity was a predictor of colony death, not health. In contrast, dying colonies had a significant spike in solidity at about 12 hours due to contraction and rounding of the colonies (B label in Fig 4E). This was followed by a drop that reached a minimum at 30 hours (C label in Fig 4E), when death occurred and extrusion of dead cells caused the convex hull to be less circular. Graphs of solidity can be used to identify at 12 hours, colonies that will die by 48 hours.

### Colony Brightness Identifies Dying Cells

As cells within a colony die, they are extruded to the top of the colony where their brightness increases. The white areas in Fig 4F are dead cells on a healthy colony at the end of incubation. Significantly more dead cells were present on the unhealthy (Fig 4G) and dying colonies (Fig 4H). To quantify dead cells on top of colonies, a bright-to-total area ratio feature was used. This feature measured the number of bright pixels in the colony as a ratio to the total area and is an indicator of cell death. All groups exhibited an increase in bright-to-total area ratio during the first 6 hours when the colonies contracted (Fig 4I), after which the bright-to-total area ratios of healthy and unhealthy colonies decreased and the ratio for the dead colonies increased up to 16 hours and stayed elevated.

To compare the progression of colony brightness over time, a minimum intensity feature (lowest pixel intensity in the colony) was monitored (Fig 4J). Throughout incubation, healthy colonies displayed a lower minimum intensity than the unhealthy and dying colonies. These data support the idea that the unhealthy and dying colonies failed to spread as well on Matrigel as healthy colonies.

To confirm cell death, colonies were labeled with Magic Red which detects activated caspases 3&7, biomarkers for apoptosis. As shown by the red staining in Fig 4K and 4L, unhealthy colonies exhibited more caspase 3&7 activity than the healthy colonies.

### 3D Visualization of Features and Custom Features Enhance Data Mining

To mine additional biological information such as correlation of features, StemCellQC can plot features against each other and play the plot as a video over time (S1 and S2 Videos). In Fig 5A, perimeter and average intensity, when plotted against each other, showed an inverse relationship (indicative of dead cells). In Fig 5B, area and the mean-squared displacement features were plotted against each other to highlight individual colonies with elevated MSD values (mainly colonies from the unhealthy group). This type of analysis can also reveal outlier colonies within a group. In addition, StemCellQC is able to plot mathematical equations using the original 24 features. In Fig 5C, a user derived equation, ratio of perimeter to the number of protrusions, is plotted. This plot displays an estimate for the average length of a protrusion for each class and shows that the protrusions on dying colonies are about twice as long as those on healthy/unhealthy colonies (Fig 5C).

### Classification Results

The input values used by the classifiers were the mean slopes of each feature. The nine individual features found by user-interpreted feature selection were tested separately giving each feature a classification rate (Table 1). Area was the best individual feature at predicting health with a 94% accuracy when using any classifier. By combining features that are not related to the same process, accuracy increased. When the number of protrusions and minimum intensity were combined, the system’s ability to distinguish hESC colony health improved to 97% accuracy when using any classifier. Results for feature selection algorithms (CFS, ChiSquare and QPFS) were also shown. All three had at least 91% accuracy and CFS was 96.47% accurate with KNN.

The classifiers were also run with the first 36, 24, and 24 hours, which are shown in Tables 2–4. For 36 hours (Table 2), area was the best feature with 88% accuracy, and combination of features improved results to 96.47%. For 24 hours (Table 3), area was still the strongest feature with 83.35% accuracy and a combination improved results to 91.17% accuracy. For 12 hours (Table 4), however, total distance travelled is the strongest feature with 71.76% accuracy. It should be noted that for 12 hours most individual features performed at about 50–60% accuracy which is slightly better than chance, however when we combine features, we are able to improved classification to 80.59% accuracy. Judging colony health by eye after a mere 12 hours of time is biased and difficult, making an 80.59% classification rate very useful. These tables show that while a certain combination of features work best using the full 48 hours of time, another combination may produce a more accurate classification if less time is used. With shorter video duration, there is an increase in accuracy using a combination of features; whereas, with longer durations, a single strong feature (such as area) can be sufficient to get accurate results.

## Discussion

StemCellQC is an innovative, cost effective, non-invasive software tool that utilizes bioinformatics to automatically monitor dynamic cell processes, cell morphology, and cell health during passaging, culture, expansion, maintenance, or experimental treatment of pluripotent stem cells. StemCellQC eliminates the need for labeling with dyes or fluorescent probes and eliminates tedious manual classification, which significantly decreases analysis time and classification errors due to observer bias.

Graphical plots of features provide quantifiable, real-time data on living hESC and are excellent analytical tools for comparing features across treatments and cell types. The plots can help users visualize trends or features that are not easily detectable by manual inspection. Cell process analysis is especially valuable in toxicological or drug studies as it provides insight into the mode of action of the treatment. For example, smoke treatment inhibited growth (area, perimeter, minor axis and protrusions), increased motility (change in centroid, total displacement, total distance traveled and MSD), and increased apoptosis (solidity and intensity features). Chemical treatments other than cigarette smoke may affect other features, and in such cases, other cell processes could be revealed by feature analysis. Multiplexing cellular process information (colony growth rate, motility, and apoptosis) increases the power of analysis, and in toxicological studies, this greatly increases the probability of detecting an effect if one exists.

StemCellQC can plot user-derived equations of features (for example: perimeter/number of protrusions) for customized types of analysis. The software can also create videos of various features plotted against each other. These plots enable correlation between features and help determine how biological processes are related over time. For example, an inverse relationship was found between colony size and colony brightness (dead cells on a colonies’ surface).

Feature analysis, when combined with a classifier, enabled identification of healthy, unhealthy, and dying colonies. Area, which classified with 94% accuracy, was the strongest feature for predicting colony health. Changes in area are not always detected by human observation, especially when colonies do not die but experience stunted growth. In clinics or research laboratories, a decrease in growth rate may signal a problem with the culture or cell quality, and this would be rapidly detected in cultures monitored using StemCellQC. While smoke treatment slowed colony growth, factors that increase growth rate may be equally important and detectable by StemCellQC. For example, when chromosomal translocations occur in hESC, growth can be accelerated [47], and this would not be desirable in clinical or research labs. Combinations of features successfully increased the accuracy of classification of unhealthy/dying colonies to 97%. Depending on the rigor needed, change in area by itself will usually be sufficient to distinguish healthy from unhealthy/dying colonies.

**Fig 6:**
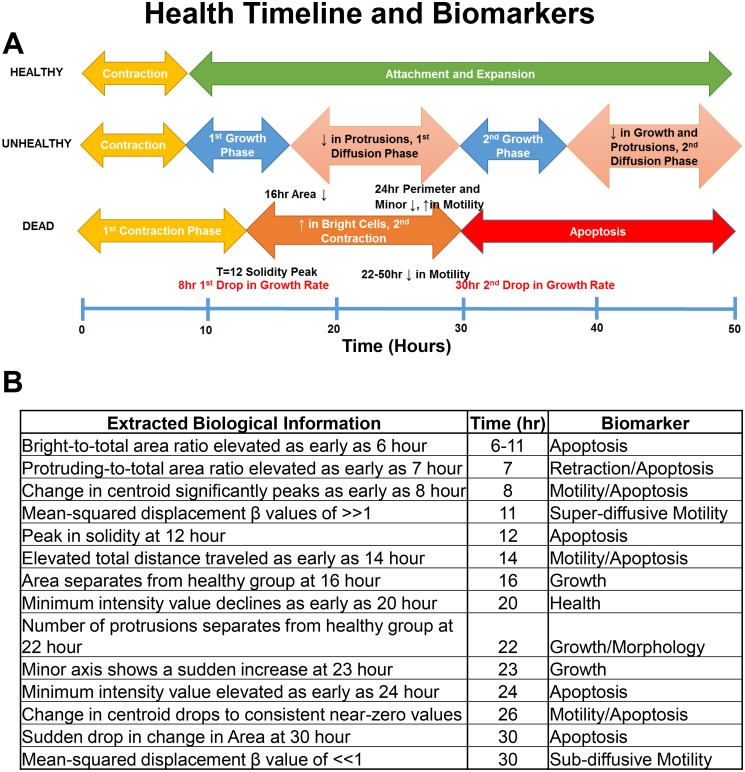
hESC Health Timeline and Biomarkers. (A) Changes in several feature values and biological events during 48 hours of incubation for healthy, unhealthy and control groups. This type of plot can be used to compare events in different groups. (B) Biomarkers that can be used to identify healthy, unhealthy, and dying colonies and their earliest detection times.

By comparing changes in features over 48 hours, biomarkers that predict biological outcomes were found at early time points (Fig 6). For example, growth rate separated dying from healthy/unhealthy colonies by 16 hours and further separated healthy from unhealthy colonies by 26 hours (Fig 1D; Fig 6A and 6B). Similar distinctions can be made from graphs for other growth features (perimeter, minor axis, and number of protrusions) (Figs 1F and 1G and 2D and 2E). Change in centroid was the strongest motility biomarker which cleanly separated healthy from dying colonies as early as 8 hours (Fig 3D). Solidity successfully separated dying colonies from healthy/unhealthy by 12 hours when used with Otsu’s segmentation (Fig 4E), and bright-to-total area ratio separated all three groups from each other by 14 hours (Fig 4I). The biomarkers for dying colonies are powerful tools for monitoring apoptosis in living cultures without use of labels or probes, which themselves often produce unwanted effects.

## Conclusions

StemCellQC is a versatile toolkit for analyzing cell processes, evaluating cell quality, and discovering biomarkers. It is designed for use with pluripotent stem cell colonies in culture, and is adaptable to other cell types. It can be used retrospectively or on-the-fly to solve numerous problems. There are at least four applications for StemCellQC. *First*, core facilities that culture pluripotent cells for distribution to research labs could monitor cell quality using non-invasive morphological tools to guarantee that distributed cells meet an acceptable uniform standard from day-to-day. This is especially important when the results of a research study may ultimately affect a patient’s health. *Second*, StemCellQC can serve as a quality control tool in future clinics that deliver therapies based on pluripotent stem cells. Such clinics will need to maintain and differentiate cells that meet future FDA criteria for transplantation to patients. A record of cell quality produced by StemCellQC would be an important part of a patient’s medical record, and could be mined after cell transfer to patients to better understand those features that work best for patient treatment. *Third*, hPSC can differentiate into specific cell types that can be used for studying genetic disorders, such as Huntington’s disease [48]. StemCellQC can monitor the behavior of cells/colonies in disease-in-a-dish models to determine how cells respond to drug treatments [49]. *Fourth*, StemCellQC could be used in laboratories that perform drug testing or that monitor chemical toxicity. Multiplexing data enhances the discovery of toxicants and biomarkers. hESC provide an excellent model for prenatal development, a process that cannot be studied experimentally in humans [20] and which is generally sensitive to environmental chemicals [50].

We are currently using StemCellQC with other pluripotent cell types and experimental conditions and found that it performed very well. We have found clear cut effects on processes such as growth, motility, death and morphology using StemCellQC with cells grown in optimal and suboptimal media, indicating StemCellQC will be useful for recognizing culture conditions that are not satisfactory. As more treatments are used, we anticipate that other processes or effects may be observed. In the future, StemCellQC software can be enhanced by adapting it to single cells and including features that correlate to cell processes such as stress, differentiation, and pluripotency. More classifiers can be added, and additional biomarkers will likely be discovered with new applications of the software.

## Supporting Information

S1 Fig (A) Diagram showing workflow used to develop StemCellQC™. (B) Diagram showing feature selection methods for classification.
(TIF)

S2 Fig Decision tree showing method for classifying hESC colonies into healthy, unhealthy or dying groups. Red arrows show decisions resulting in classification of a colony as unhealthy or dying, green arrows show decisions resulting in classification as healthy, and black arrows indicate points where the classification process was continued.
(TIF)

S3 Fig Ground truth verification of colony segmentation using ImageJ to manually segment 6 representative healthy, 6 unhealthy, and 6 dying colonies. (A, B) Normalized area and perimeter values for healthy colonies extracted by StemCellQC compared to ground truth using ImageJ. 2-way ANOVA revealed no significant differences. (C, D) Normalized area and perimeter values for unhealthy colonies extracted by StemCellQC compared to ground truth using ImageJ. 2-way ANOVA revealed no significant differences. (E, F) Normalized area and perimeter values for dying colonies extracted by StemCellQC compared to ground truth using ImageJ. 2-way ANOVA revealed no significant differences, except for a portion of the normalized area of dying colonies. This corresponds with slight over-segmentation of software due to detection of cellular debris ejected from dying colonies after their death at 30hours (* = P < 0.05).
(TIF)

S4 Fig Relationship between features and cell processes. (TIF)

S5 Fig Visual descriptors of extracted features related to area. (TIF)

S6 Fig Visual descriptors of extracted features related to morphology and area. (TIF)

S7 Fig Visual descriptors of extracted features related to motility. (TIF)

S8 Fig Visual descriptors of extracted features related to apoptosis. (TIF)

S9 Fig List of Extracted Features and Definitions. (TIF)

S1 Video Average intensity versus perimeter running plot shown for all individual healthy (green), unhealthy (blue), and dying (red) hESC colonies. (MPG)

S2 Video Mean-squared displacement versus area running plot shown for all individual healthy (green), unhealthy (blue), and dying (red) hESC colonies. (MPG)

S3 Video Phase contrast video of a representative healthy colony with the segmentation outlined in white. (MPG)

S4 Video Protrusions feature video of a representative healthy colony with the protrusions outlined in red. (MPG)

S5 Video Bright-to-total area ratio feature video with the bright dead cells of a representative unhealthy colony highlighted in white. (MPG)

S6 Video Solidity feature video of a representative dying colony with the convex hull shown in white and the colony segmentation outlined in red. (MPG)
