# Supplementary figures and images for: Evaluating Cell Processes, Quality, and Biomarkers in Pluripotent Stem Cells Using Video Bioinformatics
Source: PLoS One. 2016 Feb 5;11(2):e0148642. doi: 10.1371/journal.pone.0148642 (PMC4743914; doi:10.1371/journal.pone.0148642)

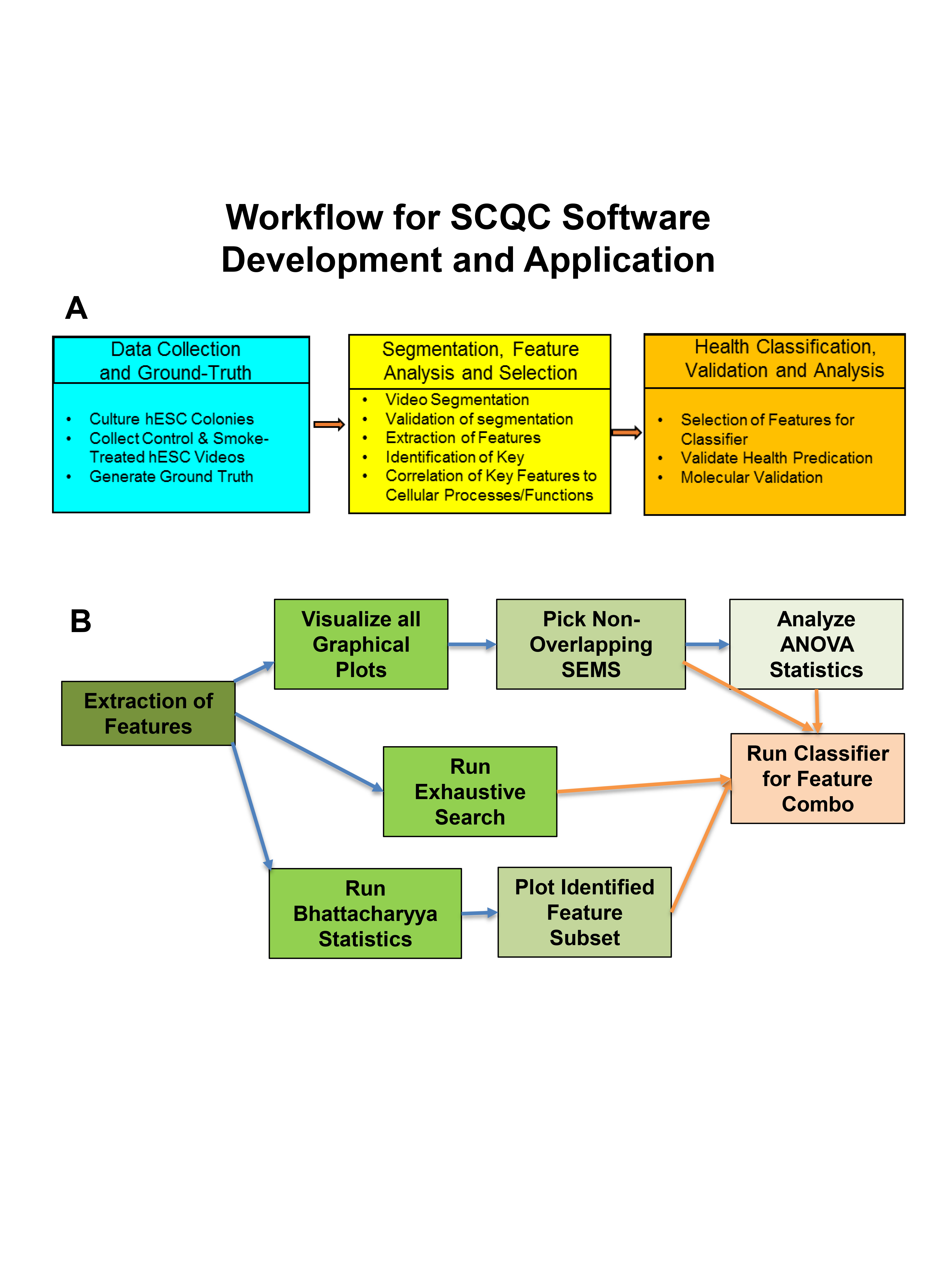

Supplement: S1 Fig — (A) Diagram showing workflow used to develop StemCellQC™. (B) Diagram showing feature selection methods for classification. (TIF) [file pone.0148642.s001.tif]

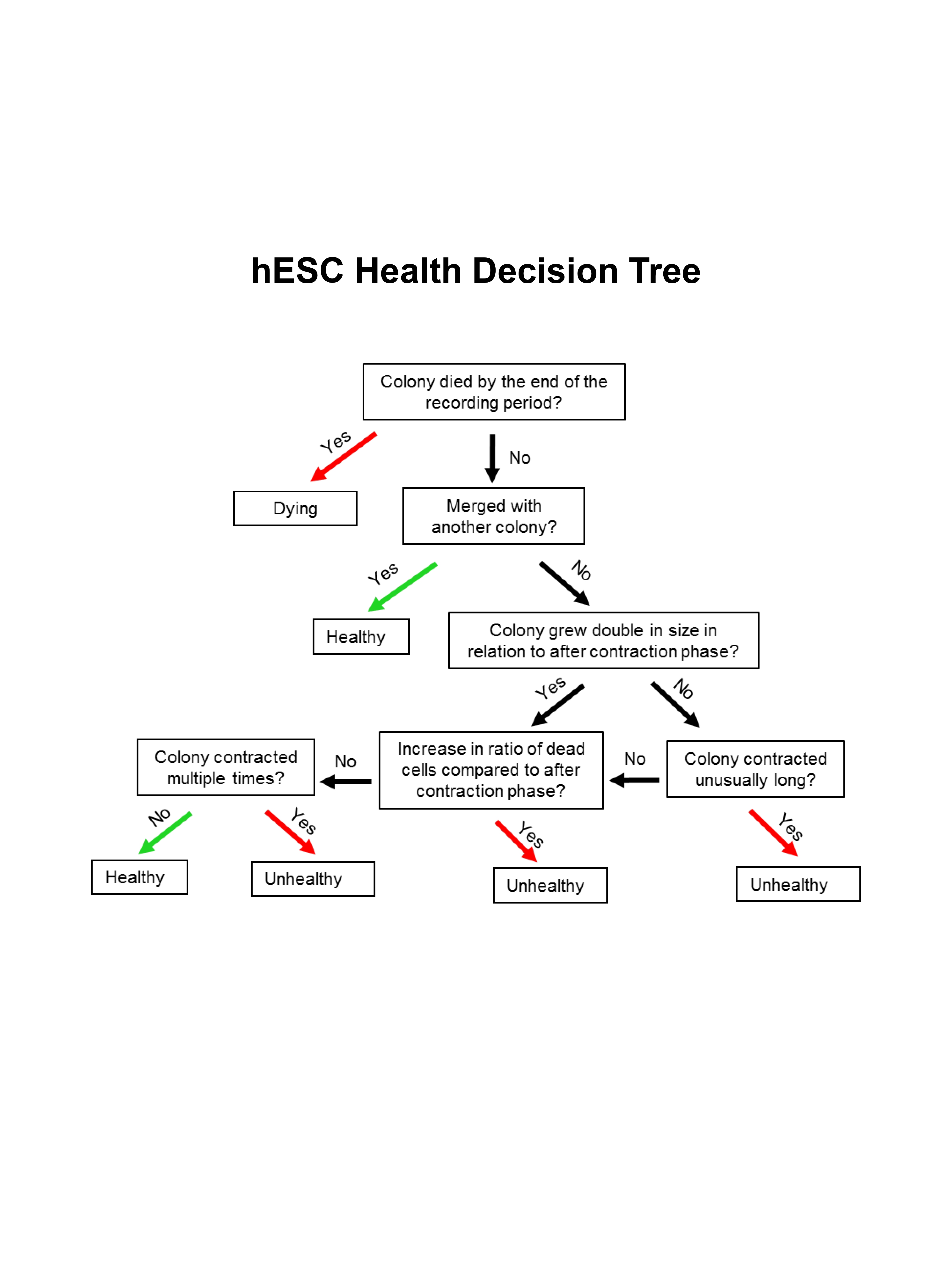

Supplement: S2 Fig — Red arrows show decisions resulting in classification of a colony as unhealthy or dying, green arrows show decisions resulting in classification as healthy, and black arrows indicate points where the classification process was continued. (TIF) [file pone.0148642.s002.tif]

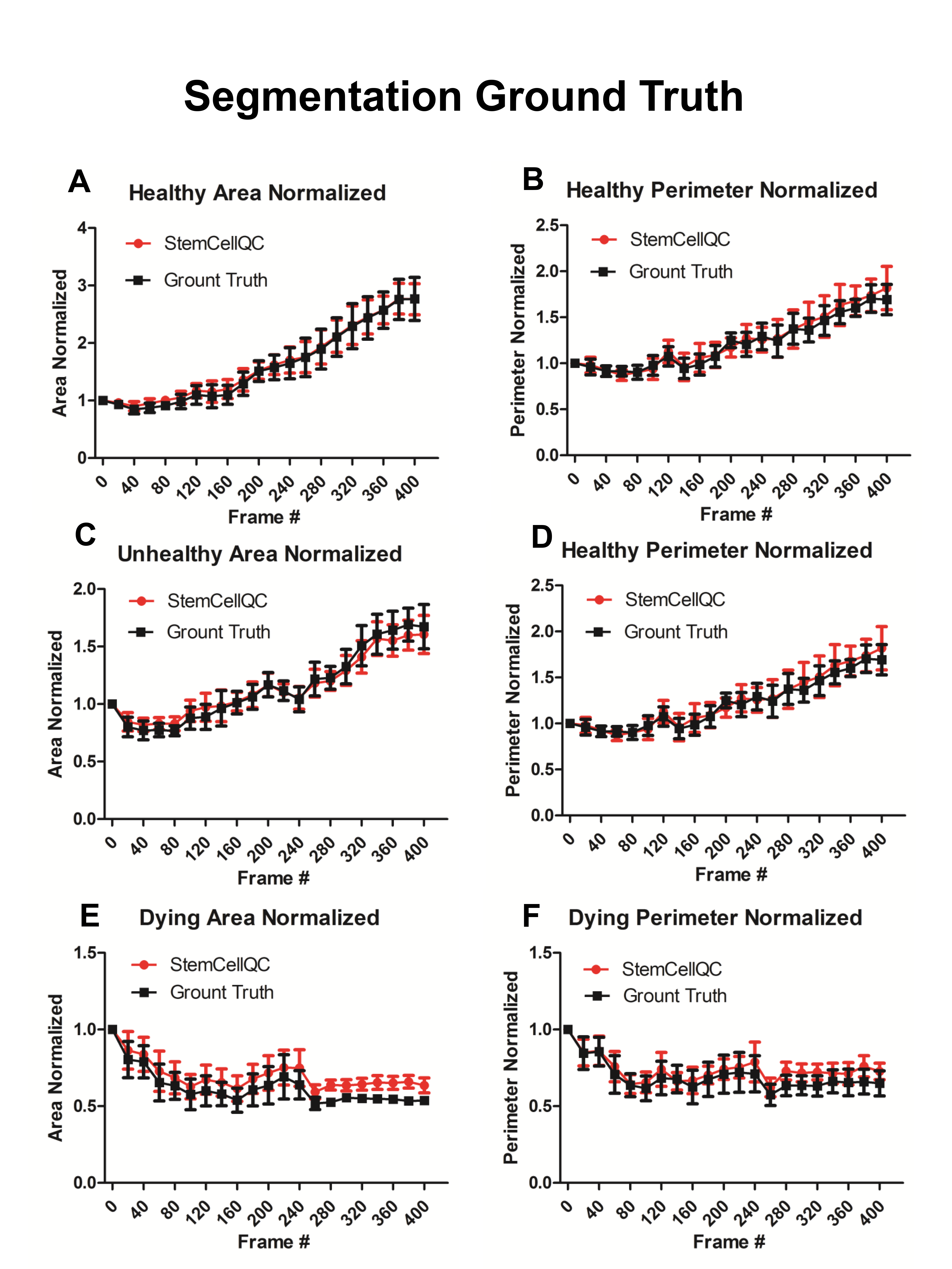

Supplement: S3 Fig — (A, B) Normalized area and perimeter values for healthy colonies extracted by StemCellQC compared to ground truth using ImageJ. 2-way ANOVA revealed no significant differences. (C, D) Normalized area and perimeter values for unhealthy colonies extracted by StemCellQC compared to ground truth using ImageJ. 2-way ANOVA revealed no significant differences. (E, F) Normalized area and perimeter values for dying colonies extracted by StemCellQC compared to ground truth using ImageJ. 2-way ANOVA revealed no significant differences, except for a portion of the normalized area of dying colonies. This corresponds with slight over-segmentation of software due to detection of cellular debris ejected from dying colonies after their death at 30hours (* = P < 0.05). (TIF) [file pone.0148642.s003.tif]

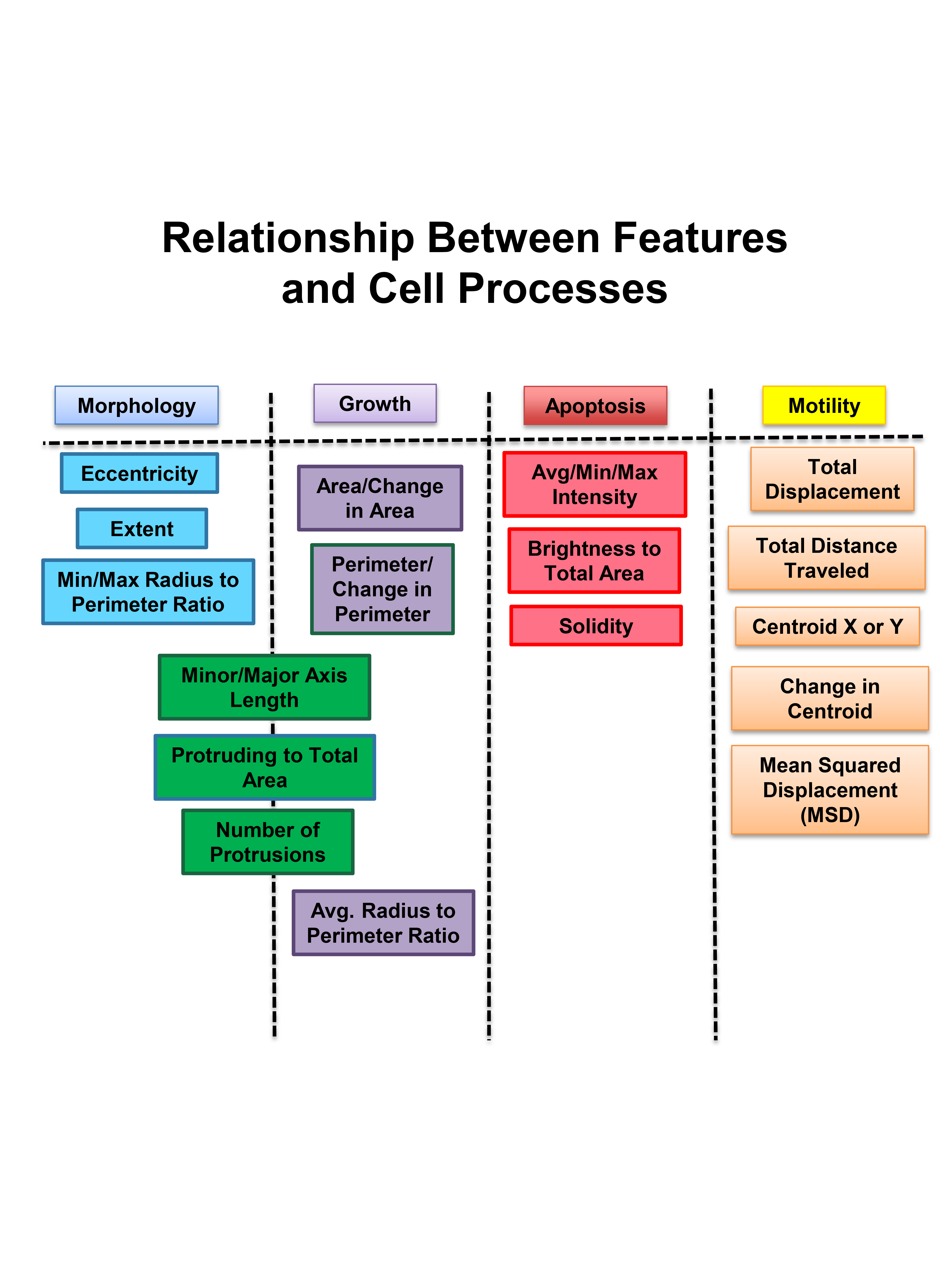

Supplement: S4 Fig — (TIF) [file pone.0148642.s004.tif]

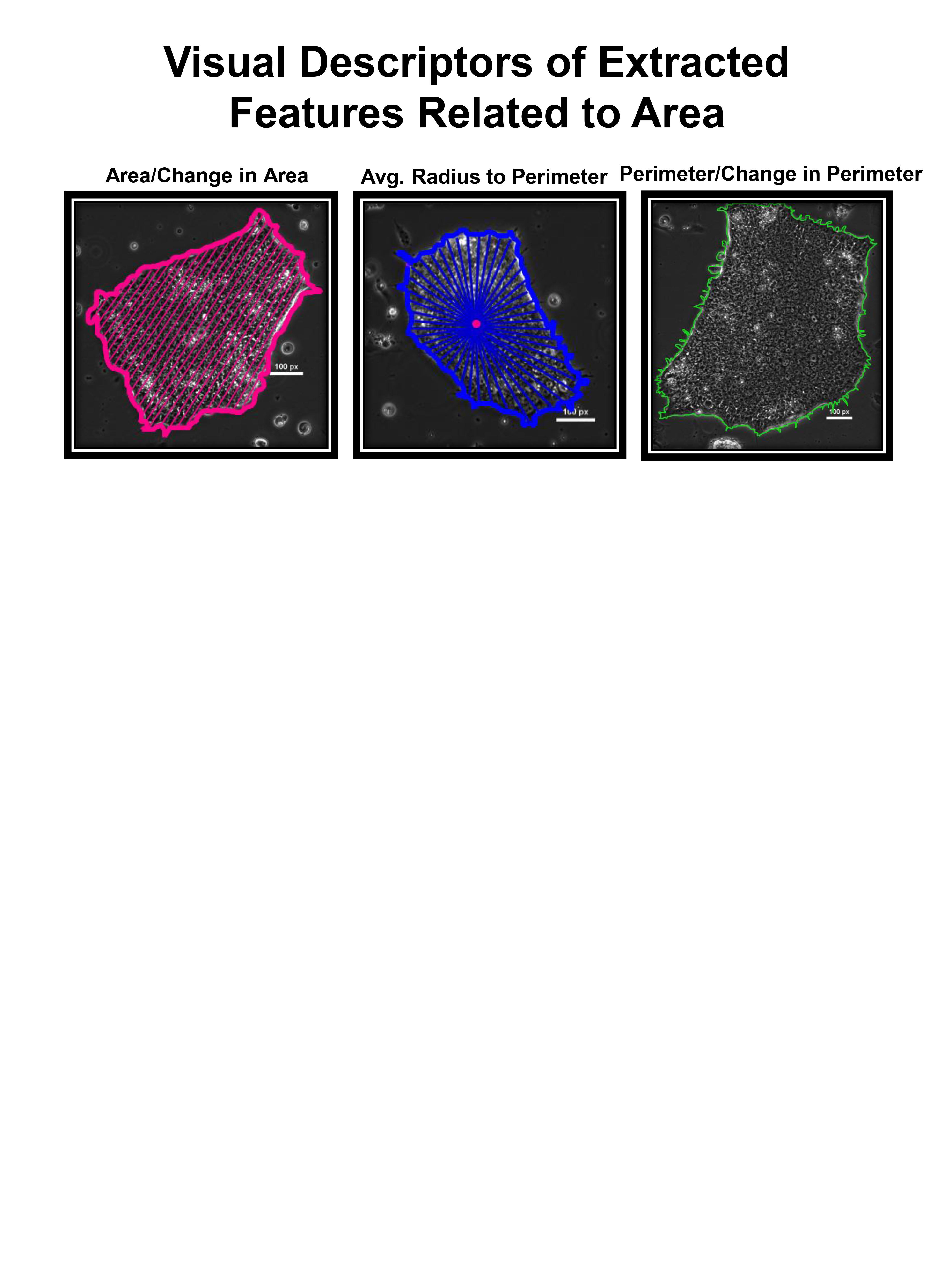

Supplement: S5 Fig — (TIF) [file pone.0148642.s005.tif]

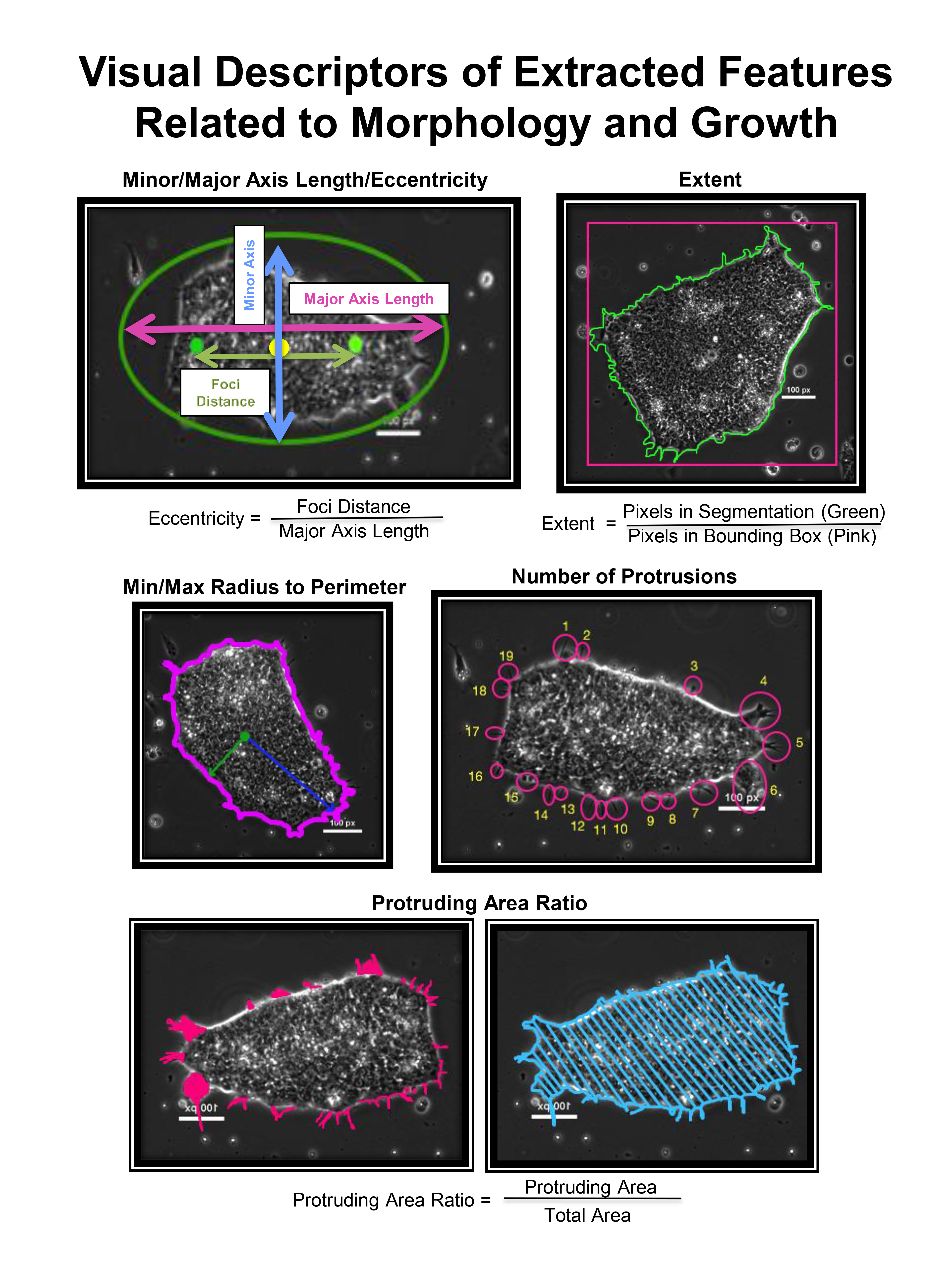

Supplement: S6 Fig — (TIF) [file pone.0148642.s006.tif]

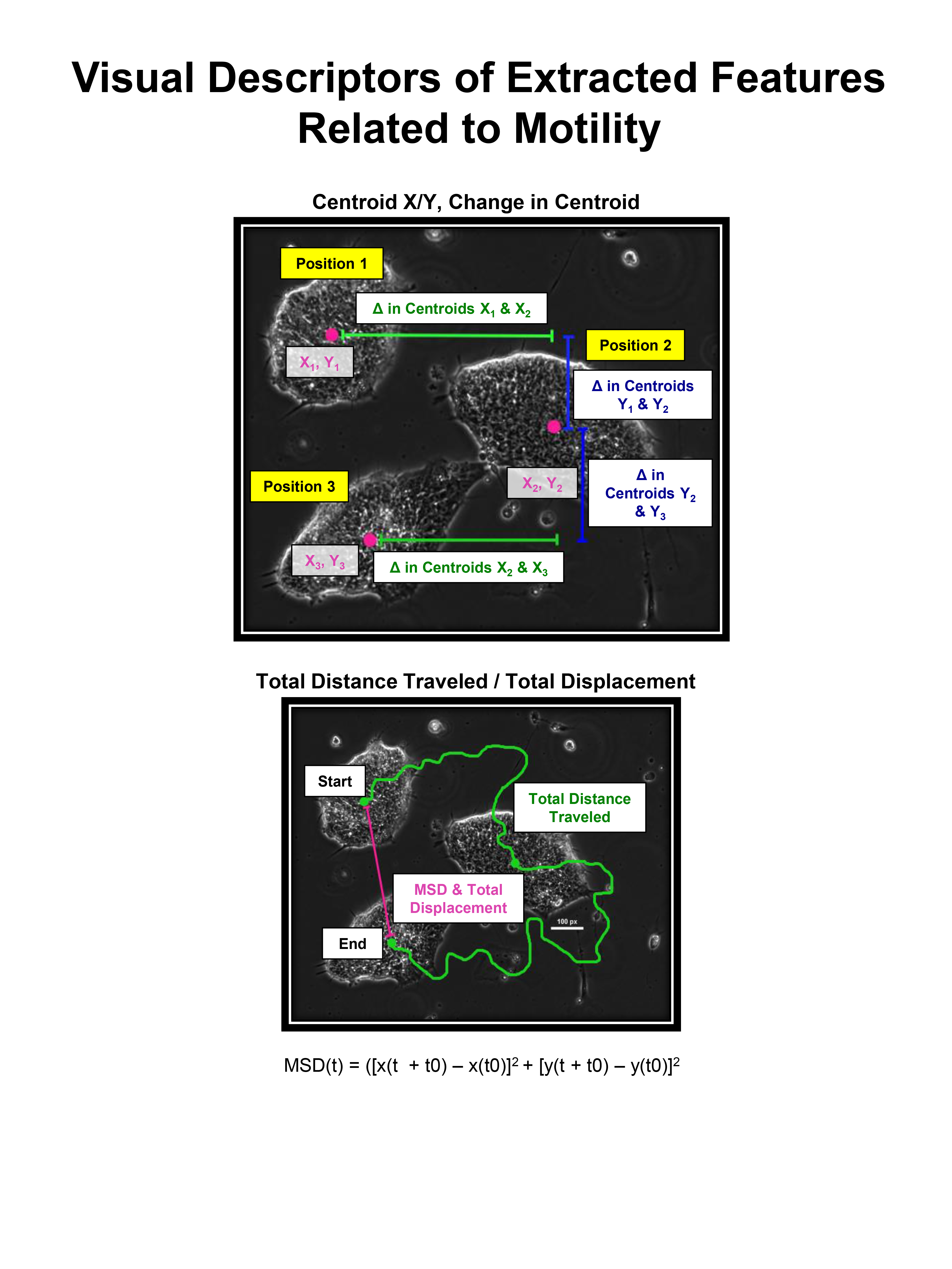

Supplement: S7 Fig — (TIF) [file pone.0148642.s007.tif]

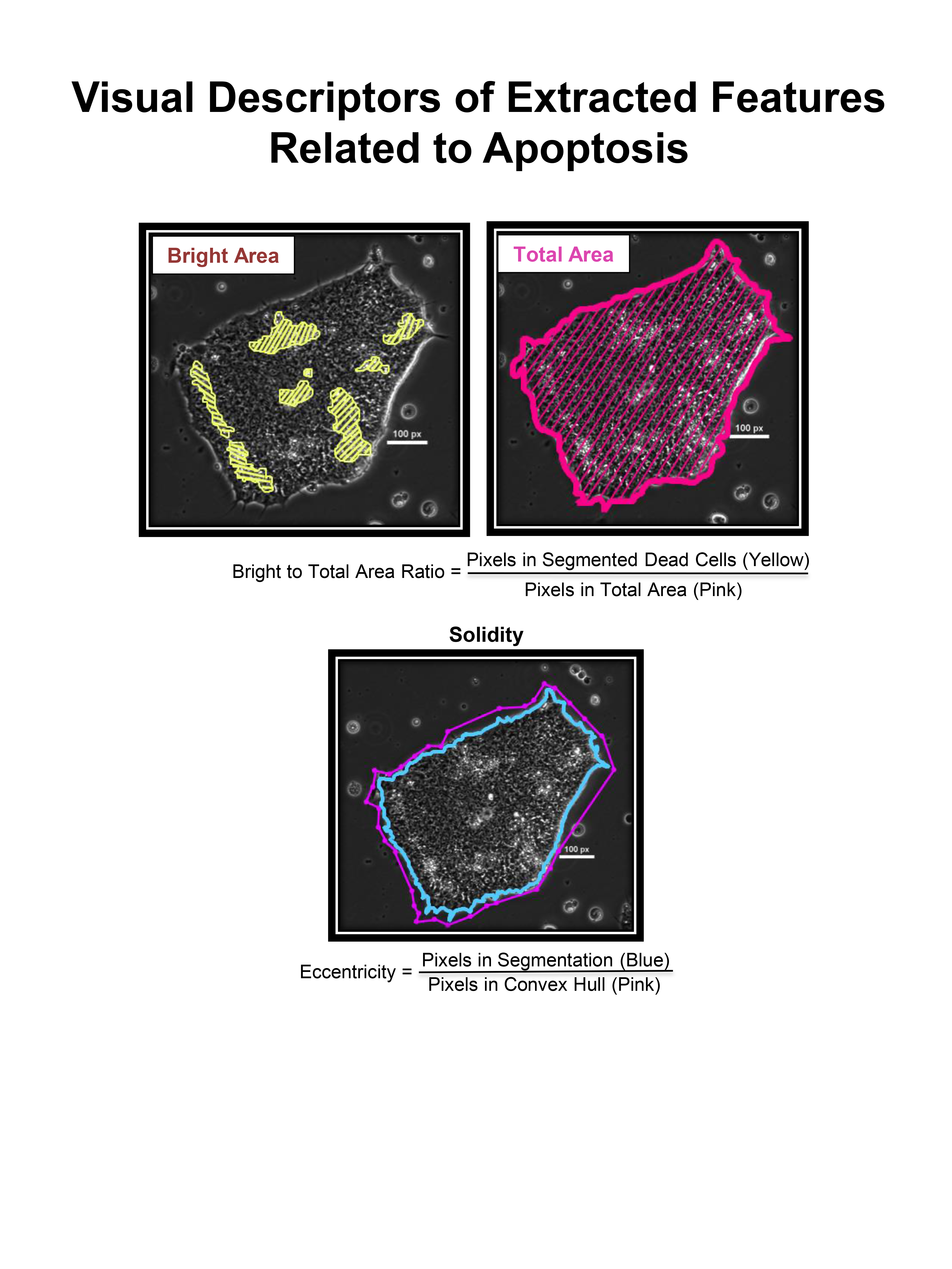

Supplement: S8 Fig — (TIF) [file pone.0148642.s008.tif]

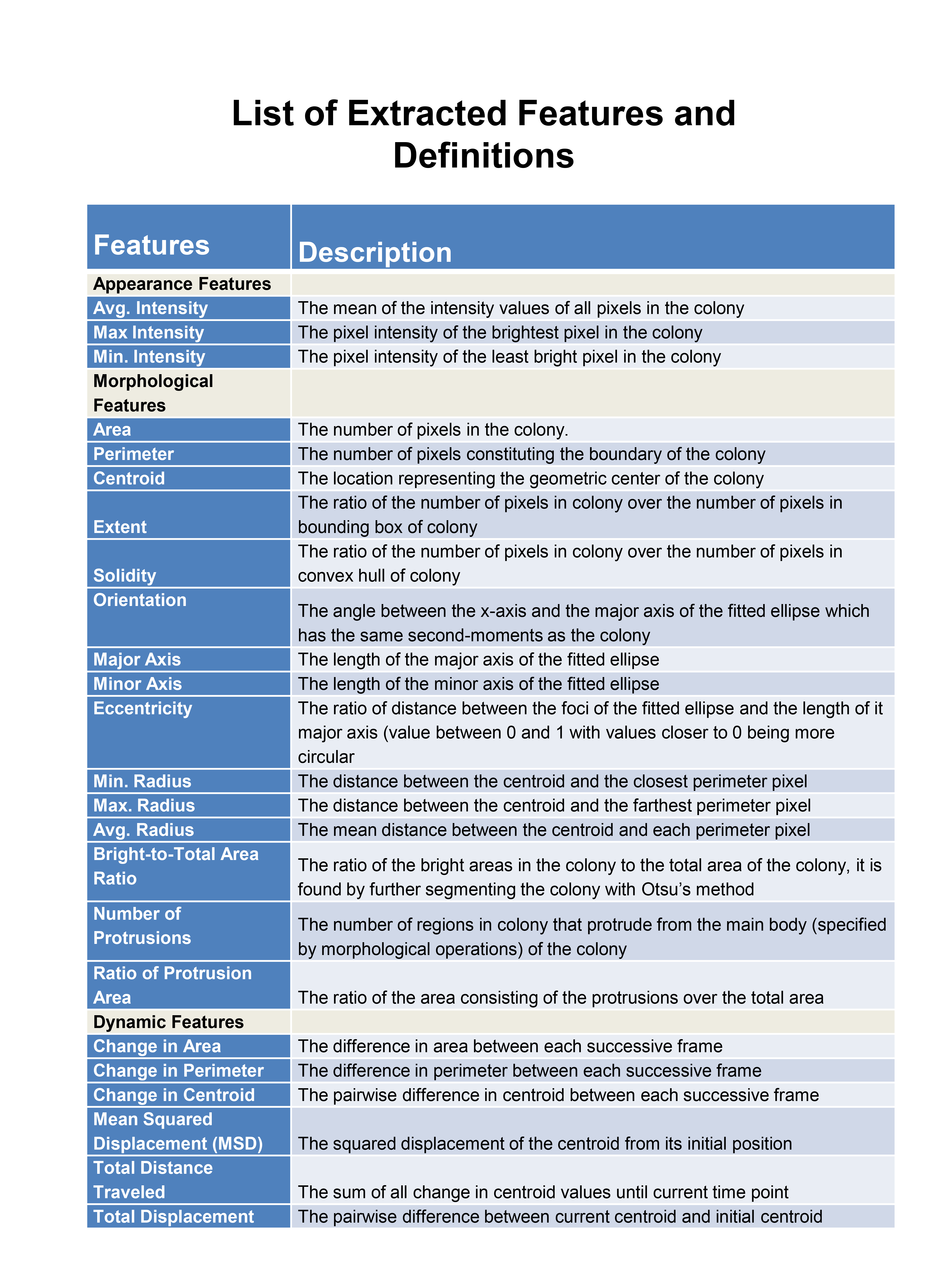

Supplement: S9 Fig — (TIF) [file pone.0148642.s009.tif]
